# Radiographic characteristics of wrists in idiopathic carpal tunnel syndrome patients

**DOI:** 10.1186/s12891-020-03254-w

**Published:** 2020-04-15

**Authors:** Kazuhiro Ikeda, Yuichi Yoshii, Takeshi Ogawa, Tomoo Ishii

**Affiliations:** 1 Department of Orthopaedic Surgery, Tokyo Medical University Ibaraki Medical Center, 3-20-1 Chuo, Ami, Inashiki, Ibaraki 300-0395 Japan; 2 Department of Orthopaedic Surgery, Mito Kyodo General Hospital, Tsukuba University Hospital Mito Area Medical Education Center, Mito, 310-0015 Japan

**Keywords:** Carpal tunnel syndrome, X-ray, Ulnar variance, Volar tilt, Radial inclination

## Abstract

**Background:**

To determine the radiographic characteristics of wrists in idiopathic carpal tunnel syndrome patients, we compared the radiographic parameters of the wrists between carpal tunnel syndrome patients and non-symptomatic controls.

**Methods:**

We evaluated radiographic parameters of 94 wrists of 62 idiopathic carpal tunnel syndrome patients and 94 asymptomatic wrists of 94 controls. Carpal tunnel syndrome was diagnosed by clinical findings and nerve conduction studies. The lack of symptoms was confirmed with the medical records and interviews for the controls. X-ray images of the postero-anterior and lateral views of the wrist were taken. Using the obtained X-ray images, the indices of radial inclination, volar tilt, ulnar variance, and transverse and antero-posterior diameters of the wrists were measured. Two raters independently performed the measurement. One rater measured without information of clinical symptoms. Inter-rater reliabilities for each parameter were evaluated by the intra-class correlation coefficients. The averages of the measurements of two raters were compared between the carpal tunnel syndrome patients and the controls.

**Results:**

The intra-class correlation coefficients were 0.58 for radial inclination, 0.77 for ulnar variance, 0.99 for transverse diameter, 0.60 for volar tilt, and 0.91 for antero-posterior diameter. Statistically significant correlations were found for all parameters (*P* < 0.01). The ulnar variance was significantly larger in the carpal tunnel syndrome patients compared to the controls (1.7 +/− 1.8 mm and 0.8 +/− 1.5 mm for the patients and controls, respectively *P* < 0.01). There were no significant differences in the other parameters.

**Conclusions:**

Significant differences in the ulnar variance were observed between carpal tunnel syndrome patients and controls. This suggests that the imbalance of radioulnar bone length is one of the risk factors to develop carpal tunnel syndrome. The positive ulnar variance may be an index that needs attention to the development of carpal tunnel syndrome.

**Level of evidence:**

level III, a case control study.

## Background

Carpal tunnel syndrome (CTS) is one of the most common peripheral neuropathies [1]. There are several etiologies of carpal tunnel syndrome including idiopathic, wrist trauma, oral contraceptives, pregnancy, arthritis, collagen-vascular diseases, diabetes, hypothyroidism, and occupations or hobbies that involve repetitive motion of the wrist [2]. When considering the risk factors of median nerve compression in the carpal tunnel, it is necessary to consider the size of the carpal tunnel and the local factor narrowing the tunnel. It has been suggested that carpal tunnel narrowness is one of the risk factors, because idiopathic CTS develops at a high rate in women with a small carpal tunnel cross-sectional area [3]. Anatomically, there are two sites of median nerve compression, i.e., the proximal edge of the carpal tunnel and the narrowest portion at the hook of hamate. Because of the changes in thickness and rigidity between the proximal portion of the flexor retinaculum and the forearm facia, changes of fluid pressure in the carpal tunnel have been reported with wrist movement [4].

It is well known that CTS develops as a late stage disorder associated with deformity healing after distal radius fracture [5–9]. Several reports have analyzed the pathogenesis of CTS following distal radius fractures and discussed predisposing risk factors [6, 8, 10–15]. It has been suggested that an abnormal course of the median nerve at the wrist or carpal tunnel volume loss due to malunion of the distal radius is one of the pathomechanisms involved in the development of CTS [3]. This suggests that morphology of the wrist joints may also play an important role for the etiology of idiopathic CTS. To know the morphology of the wrists, simple X-ray are widely used. Simple wrist X-ray can provide morphological image of wrist more easily and quickly than other radiological examinations with low cost. In addition, morphometry in plain radiographs are useful to identify the characteristics of wrist joint. If wrist morphometric parameters are related to the pathology of CTS, it may be useful in predicting or estimating the risk of carpal tunnel syndrome. However, there were few reports that evaluated the morphology of the wrist in idiopathic CTS patients. The significance of radiography in carpal tunnel syndrome has not been established. To assess the radiographic characteristics in CTS patients, we compared the roentgenographic parameters of the wrists between CTS patients and non-symptomatic controls. We hypothesized that individuals with idiopathic CTS may have differences in the radiographic findings of their wrists compared with healthy individuals.

## Methods

This was a case control study (level of evidence: level III). The study protocol was approved by our Institutional Review Board. Table 1 shows the average age and the number of male and female cases for both groups. CTS patients were diagnosed during the period from October 2011 to December 2017. The controls were randomly chosen from the list of persons who took wrist X-ray during the same period and matched the age/gender to the CTS patients. Ninety-four wrists of 62 idiopathic CTS patients (male: 42 hands, female: 52 hands) and 94 asymptomatic wrists of 94 controls (male: 40 hands, female: 54 hands) were evaluated. There were 31 CTS patients (14 male, 17 female) who had both hands symptoms. There were no significant differences between CTS patients and controls for the age distribution and gender ratio.

**Table 1:** Average age and gender of both groups

	Age	Male	Female	Total
CTS Patients	65.2±13.2	28 (42 Hands)	35 (52 Hands)	62 (94 Hands)
Control	64.1±13.9	40 (40 Hands)	54 (54 Hands)	94 (94 Hands)
*P* value	0.64	0.88		/

The patients and controls were age/gender matched

CTS was diagnosed by clinical findings such as numbness and sensory disturbance on the radial side of the ring finger from the thumb finger, and having at least one positive provocative test result (Tinel’s sign at the wrist and Phalen’s maneuver). Thenar muscle atrophy was also considered one positive finding for the diagnosis of CTS. All patients underwent nerve conduction study and ultrasound imaging. The CTS diagnosis was confirmed with clinical symptoms and meeting the criteria of nerve conduction velocity findings with the American Association of Electrodiagnostic Medicine (AAEM) guidelines [16]. CTS patients with a history of systemic disease associated with a higher incidence of CTS, such as chronic renal failure, thyroid disease, diabetes, obesity (=body mass index greater than or equal to 30), or rheumatoid arthritis, were excluded. In addition, patients with a history of any upper extremity trauma were excluded. There were no anatomical variants in the wrists or space occupying lesions in the carpal tunnel in any of the CTS patients. X-ray of the postero-anterior and lateral view of the wrists were taken for both CTS patients and controls. The control wrists which had taken the wrist X-ray for a screening test or for comparison with the symptomatic wrist, excepting the symptoms of CTS, were obtained from our medical records. The lack of symptoms were confirmed with the medical records and interviews for the controls. The controls were age and gender matched with the CTS patients. The X-rays were taken at the initial consultation for their symptoms in both groups.

**Fig. 1:**
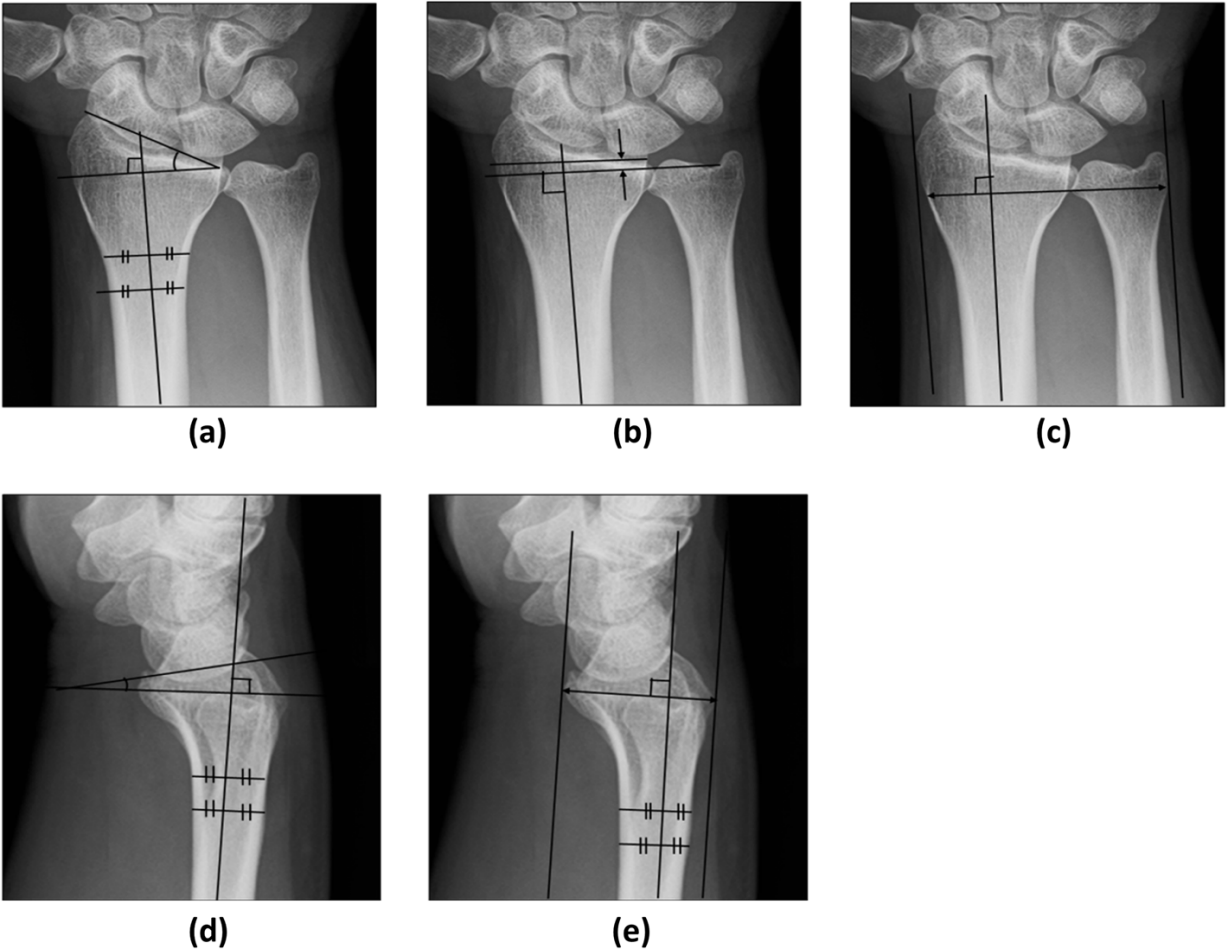
Measurements of roentgenographic indices. **a** Radial inclination was defined as the angle between one line connecting the radial styloid tip and the ulnar aspect of the distal radius and a second line perpendicular to the longitudinal axis of the radius. **b** Ulnar variance was defined as the relative lengths of the distal articular surfaces of the radius (middle line of the surface) and ulna (fovea). **c** The transverse diameter was defined as the distance between the radial edge of the radius and the ulnar edge of the ulna at the level of the radio-carpal joint. **d** Volar tilt was defined as the angle of the distal radial surface with respect to a line perpendicular shaft. **e** The antero-posterior diameter was defined as the distance between the volar and dorsal edge of the distal radius

The radiology technicians who took the X-ray images were blinded to the clinical symptoms. The postero-anterior view was obtained with the elbow flexed 90 degrees (the ulna perpendicular to the humerus) and the forearm in the pronated position. The lateral view was obtained with the elbow flexed 90 degrees and adducted against the trunk. The wrists were in a neutral position with no flexion, extension, or deviation in either view. Using the obtained X-ray images, the indices of radial tilt, volar tilt, ulnar variance, and transverse and antero-posterior diameters of the wrists were measured with image analyzing software (Synapse Vincent, Fujifilm Holdings Co., Fig. 1). The radial inclination, ulnar variance, volar tilt are most often used parameters to identify malalignment of the wrist [17]. The transverse and antero-posterior diameter were measured to estimate the carpal tunnel sizes. The angle between a line from the dorsal edge to the volar edge of the radius and the line perpendicular to the longitudinal axis of the radius was measured as the volar tilt. The angle between a line from the radial styloid tip to the ulnar aspect of the distal radius and a line perpendicular to the longitudinal axis of the radius was measured as the radial inclination. The transverse diameter was defined as the distance between the radial edge of the radius and the ulnar edge of the ulna at the level of the radio-carpal joint. The antero-posterior diameter was defined as the distance between the volar and dorsal edge of the distal radius. Two raters (A, B) independently performed the measurement. The rater B measured without information of clinical symptoms.

### Statistical analysis

The results are expressed as mean +/− standard deviation. Inter rater reliabilities for each parameter were evaluated by the intraclass correlation coefficients (ICC) for the raters A and B measurements. The parameters were compared between the CTS patients and the controls. For the normality test of datasets, the Shapiro-Wilk test was used. Welch’s t-test was applied in normal distribution indices (volar tilt, antero-posterior diameter) and Mann-Whitney’s U test was applied in non-normal distribution indices (radial inclination, ulnar variance, and transverse diameter). The averages of the measurements of the two raters were compared between the CTS patients and the controls. *P* values of < 0.05 were considered significant. The diagnostic potentials of the parameters were assessed by the receiver operating characteristic (ROC) curves. Performances of the diagnostic variables were quantified by calculating the area under the ROC curve (AUC) for the parameters. The cut-off values on the ROC curves were defined by the highest point on the vertical axis and the point furthest to the left on the horizontal axis (upper left corner). All analyses were performed using BellCurve for Excel version 2.12 (SSRI Co., Tokyo, Japan).

## Results

**Fig. 2:**
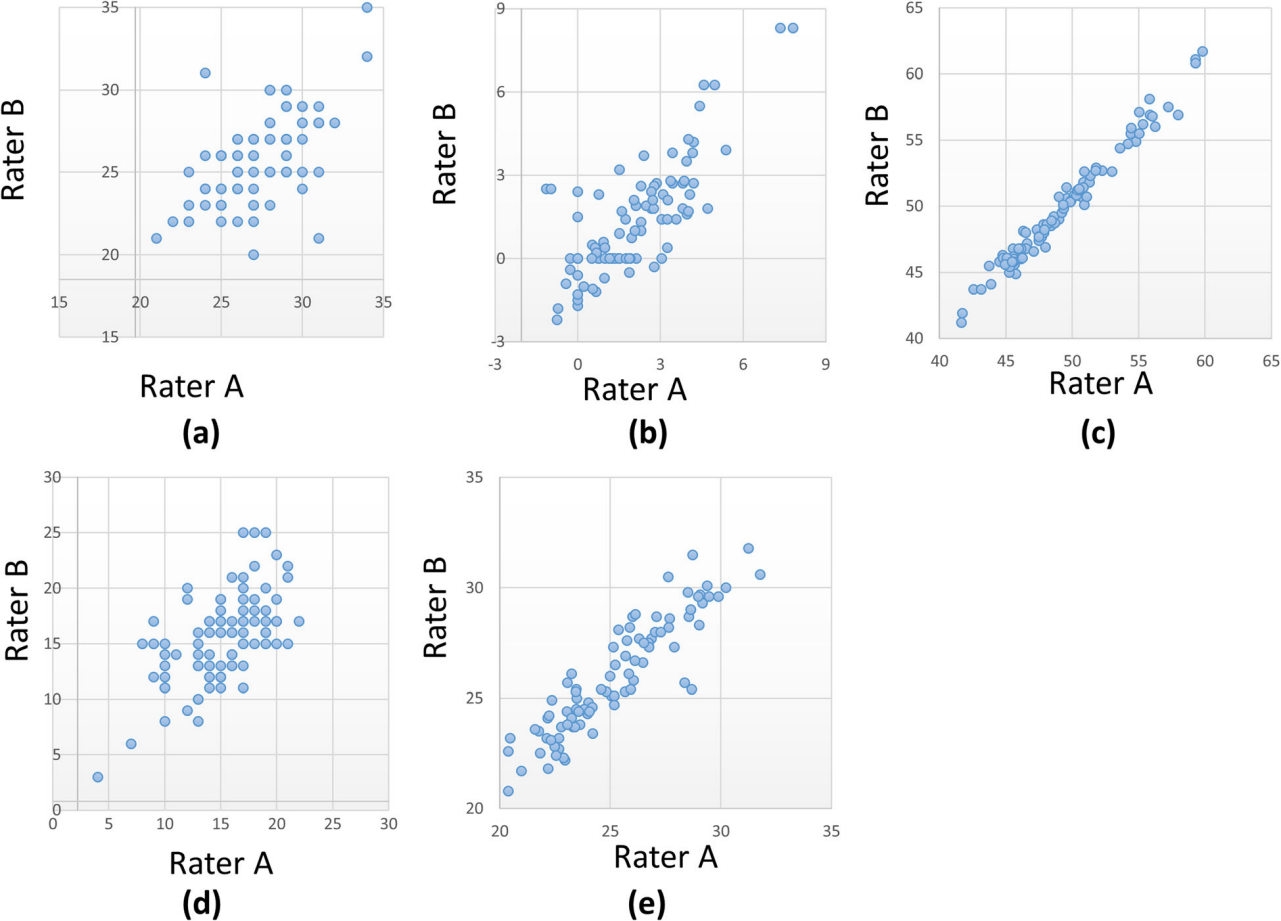
Scatter plot for the two rater measurements. **a** Radial inclination, (**b**) Ulnar variance, (**c**) Transverse diameter, (**d**) Volar tilt, (**e**) Antero-posterior diameter. Statistically significant correlations were found for all parameters (*P* < 0.01). The ICCs for the radial inclination, ulnar variance, transverse diameter, volar tilt, and antero-posteodiameter were 0.58, 0.77, 0.99, 0.60, and 0.91, respectively

The results of correlations between rater A and B are shown in Fig. 2. Statistically significant correlations were found for all parameters (*P* < 0.01). The intra-class correlation coefficients were 0.58 for radial inclination, 0.77 for ulnar variance, 0.99 for transverse diameter, 0.60 for volar tilt, and 0.91 for antero-posterior diameter. The transverse diameter showed the highest correlations. The radial inclination showed the lowest correlations.

**Fig. 3:**
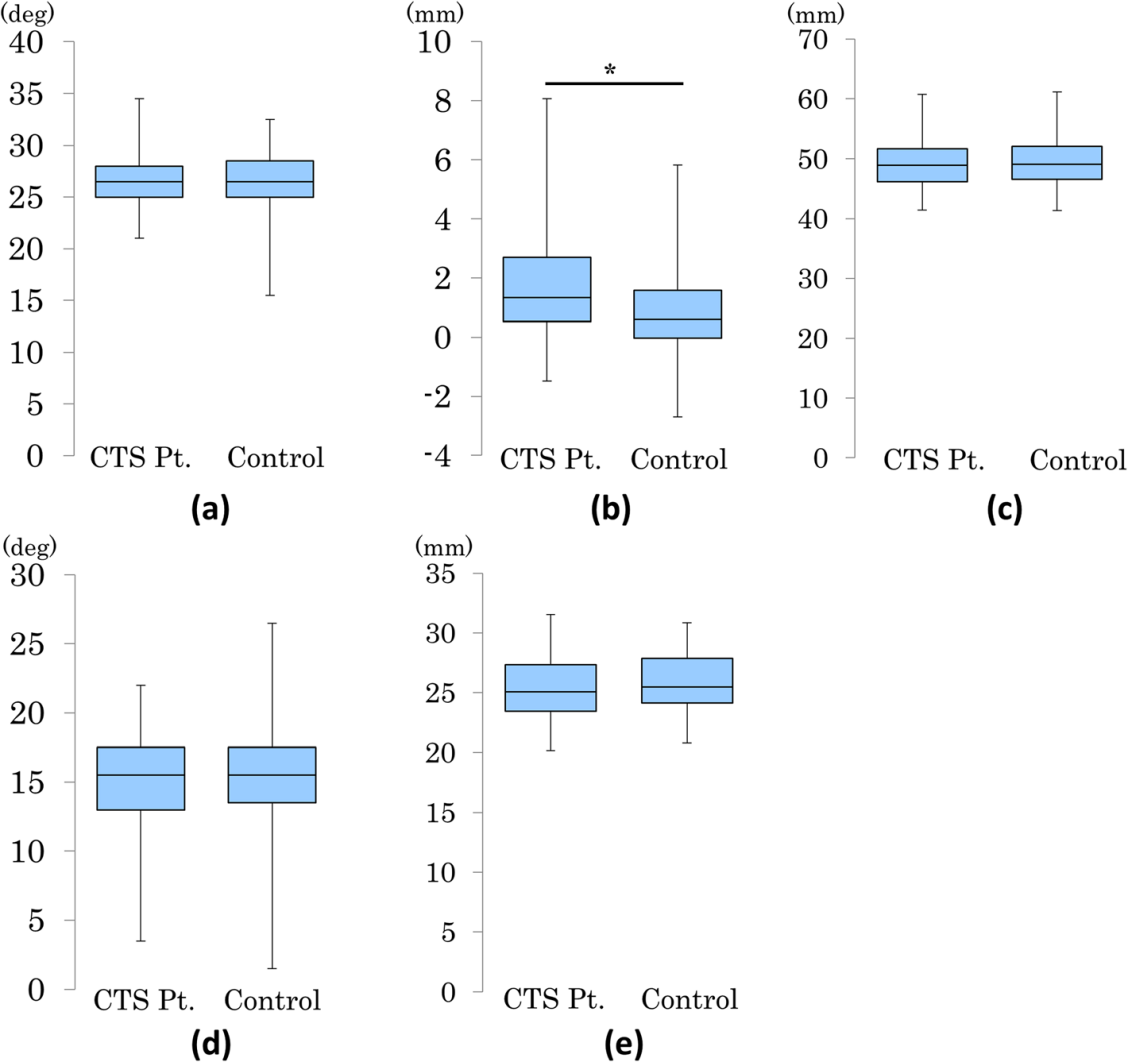
Results of roentgenographic indices. **a** Radial inclination, (**b**) Ulnar variance, (**c**) Transverse diameter, (**d**) Volar tilt, (**e**) Antero-posterior diameter. The ulnar variance was significantly larger in the CTS patients compared with the controls (*: P < 0.01)

The results of the roentgenographic indices for the CTS patients and controls are shown in Fig. 3. The radial inclinations were 26.6 +/− 2.2 degrees and 26.4 +/− 2.8 degrees for the CTS patients and controls, respectively (*P* = 0.71). The ulnar variances were 1.7 +/− 1.8 mm and 0.8 +/− 1.5 mm for the CTS patients and controls, respectively (*P* < 0.01). The transverse diameters were 49.6 +/− 4.2 mm and 49.7 +/− 4.2 mm for the CTS patients and controls, respectively (*P* = 0.80). The volar tilts were15.2 +/− 3.8 degrees and 59.8 +/− 6.4 degrees, respectively (*P* = 0.67). The antero-posterior diameters were 25.4 +/− 2.7 mm and 25.9 +/− 2.5 mm for the CTS patients and controls, respectively (*P* = 0.20). The ulnar variances were significantly larger in the CTS patients compared to the controls.

**Fig. 4:**
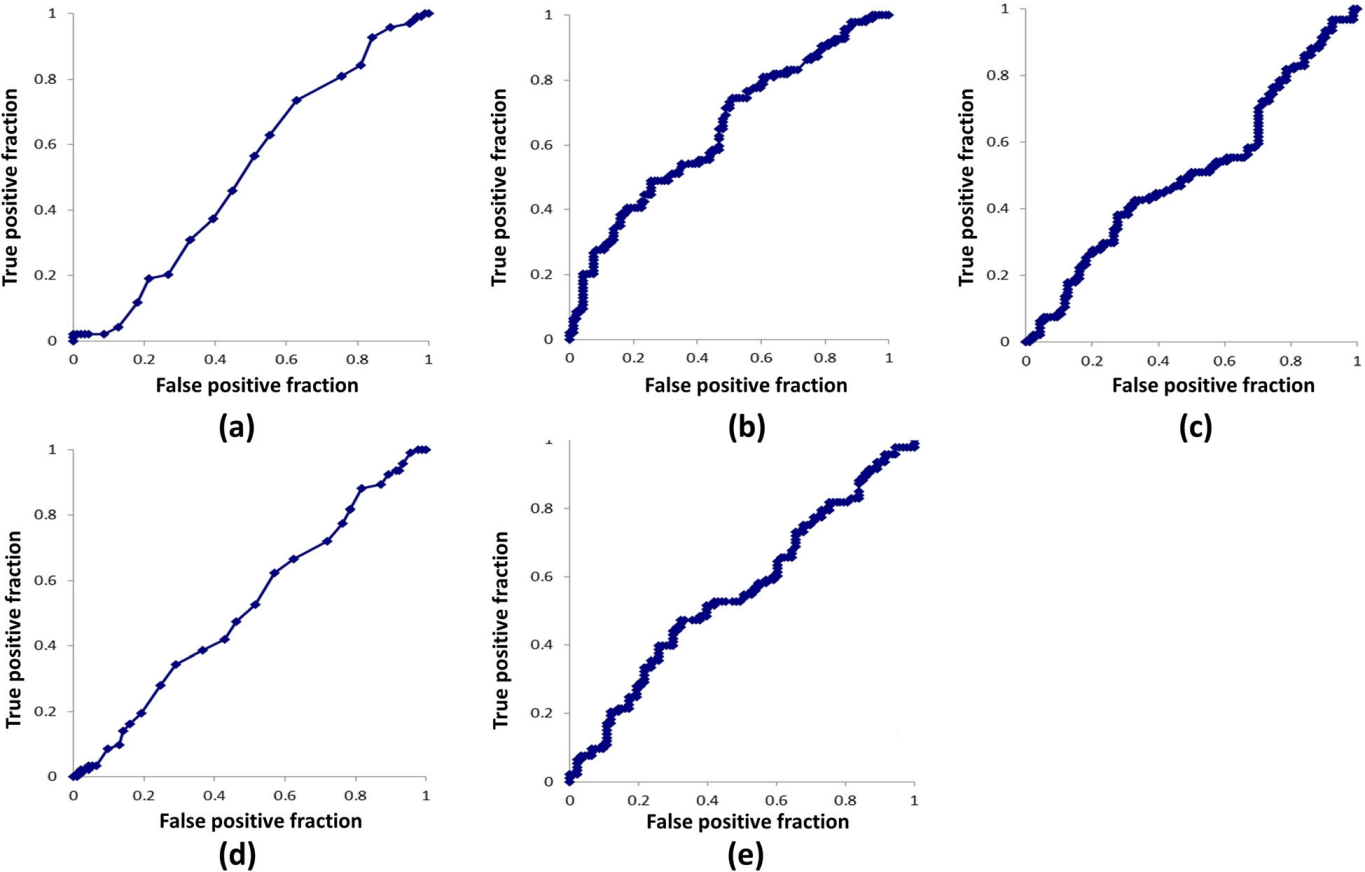
Results of ROC curve analysis. **a** Radial inclination, (**b**) Ulnar variance, (**c**) Transverse diameter, (**d**) Volar tilt, (**e**) Antero-posterior diameter

The results of ROC curve analysis are shown in Fig. 4. The cut-off values and odds ratios for each parameter are shown in Table 2. The AUC values were 0.52, 0.65, 0.51, 0.52 and 0.55 for the radial inclination, ulnar variance, transverse diameter, volar tilt, and antero-posterior diamter, respectively. The ulnar variance had the highest AUC value, and the transverse diameter had the lowest. The cut-off value to discriminate individuals at risk of CTS from the asymptomatic control was 0.58 mm.

**Table 2:** Results of ROC curve analysis

	AUC values	Cut-off values	Odds Ratio
**Radial inclination**	**0.52**	**26 deg**	**1.4**
**Ulnar variance**	**0.65**	**0.58 mm**	**2.8**
**Transverse diameter**	**0.51**	**47.9 mm**	**1.5**
**Volar tilt**	**0.52**	**16 deg**	**1.3**
**Antero-posterior diameter**	**0.55**	**24.7 mm**	**1.9**

The cut-off values on the ROC curves were defined by the highest point on the vertical axis and the point furthest to the left on the horizontal axis (upper left corner)

## Discussion

Several factors are involved in the onset of idiopathic CTS. Mechanical stress to subsynovial connective tissue accompanying flexion-extension of the wrist or fingers is considered to be a leading factor for the development of CTS [18]. Natural carpal tunnel narrowness is presumed to be another risk factor [3]. The other reports showed no significant differences in the carpal tunnel cross-sectional area between idiopathic CTS patients and healthy controls [19, 20]. It is well known that CTS develops as a late stage disorder associated with malunions of distal radius fractures [8, 10, 13, 21]. This suggests that the morphology of wrist joints (radiocarpal, ulnocarpal and distal radioulnar joints) may affect the development of CTS.

The usefulness of plain radiography for the diagnosis of CTS was uncorroborated. The advantages of taking X-ray are simple, quick, possibility of multiple inspections, and low cost. The disadvantages of the X-ray is that it is unable to see the soft tissues. As it cannot reveal the abnormalities in the soft tissues that constitute carpal tunnel, plain radiography had been considered to have a limited role for the diagnosis of idiopathic CTS [22–24]. It could be useful only in cases associated with bony stenosis, fracture and soft tissue calcification. There was a report showing only 0.4% of cases with specific findings in plain radiograph related to treatment policy [24]. Therefore, it was suggested that plain radiograph should not be taken as a routine CTS diagnostic tool unless hard tissue-related changes were suspected. Recent advances in imaging techniques in ultrasound and MRI, i.e., shear wave elastography [25], pressure-monitor elastography [26], diffusion tensor imaging of MRI [27], have been reported to be more beneficial in diagnosing neurological conditions of carpal tunnel syndrome.

To date, there are few studies which showing the significance of X-ray measurements for the assessment of CTS. The reasons for measuring the indices of radial inclination, volar tilt, ulnar variance, and transverse and antero-posterior diameters, are their widespread use and simplicity/reproducibility of measurements. If the size of the wrist joint is small, it is expected that the carpal tunnel would be small. However, the wrist size parameters of antero-posterior diameter/ transverse diameter did not differ between the groups. On the other hand, there was a significant difference in the ulnar variance between CTS patients and controls. This suggests that carpal tunnel syndrome was not due to native bony carpal tunnel narrowness, but rather due to imbalance of distal radio-ulnar joint. The uniqueness of this study was that it could reveal the difference between CTS patients and controls in one morphological parameter of the wrist. The positive ulnar variance may be recognized as one of the risk factors of developing CTS with larger studies. In a study of CTS with distal radius malunions, the residual dorsal angle of the distal radius had a statistically significant correlation with the occurrence of CTS [15]. The carpal tunnel is narrowed with volar flexion of the midcarpal joint, dorsiflexion of the lunate, shortening and dorsal tilt of the radius [10, 22]. More than 60% of wrists in CTS after distal radius fracture had unacceptable deformity of the distal radius [7]. Due to the distal radius malunion, the median nerve is placed in a narrow carpal tunnel and abnormal pathway. This suggests that the anatomical configuration of the proximal carpal tunnel due to the distal radius shape may be one of the main causes of CTS. Therefore, we expected that the volar tilt and radial inclination would be different when comparing between CTS patients and controls. However, there were no significant differences in these parameters. This suggests that the volar tilt and radial inclination in normal range would not be a risk of development of CTS.

The biomechanical conditions of positive ulnar variances is similar to the wrist changes in the CTS after distal radius fracture. It has been thought that shortening of the radius lead to narrowness of the carpal tunnel [9, 28]. Normally, 80% of load applied to the wrist were transmitted through radius, and 20% were transmitted through ulna. The positive ulnar variance will increase the load bearing by the ulnocarpal joint [29]. In addition, there was a report which showed a positive relationship between decreased cross-sectional area around the distal radioulnar joint and positive ulnar variance on radiologic investigation [30]. Although there is no direct relationship between positive ulnar variance and median nerve compression, the positive ulnar variance may affect the development of CTS through the load on the ulnocarpal joint.

There were several limitations in this study. First, the definition of normal controls needs further consideration. We selected X-rays of asymptomatic wrists showing no symptoms at the time of the hospital visit. It is uncertain as to whether the control subjects will develop CTS in the future. We enrolled control subjects from among individuals visiting the hospital, who may not be representative of the general population. Second, there is only one X-ray taken per patient. To assess the reproducibility of each measurement, it may have been necessary to evaluate at multiple time points. Finally, since each measurement value for both groups was within the normal range, the parameters showing significant differences between CTS and controls are useful as a relative index.

## Conclusions

The knowledge of a particular, quantifiable, predisposing factor for CTS would be of great interest for prevention and early diagnosis. Our results suggest that the imbalance of radioulnar bone length is one of the risk factors to develop carpal tunnel syndrome. The positive ulnar variance may be an index that needs attention to the development of carpal tunnel syndrome.

## Data Availability

The datasets analyzed during the current study are available from the corresponding author on reasonable request.

## Abbreviations

CTS: Carpal tunnel syndrome
AAEM: American association of electrodiagnostic medicine
ICC: Intraclass correlation coefficients
ROC: Receiver operating characteristic
AUC: Area under the ROC curve
